# Lower glomerular filtration rate after mild stroke induces cognitive impairment by causing endothelial dysfunction

**DOI:** 10.1038/s41598-024-57444-w

**Published:** 2024-03-23

**Authors:** Xu Yan, Huan Chen, Xiuli Shang

**Affiliations:** 1 Department of Neurology, The First Affiliated Hospital of China Medical University, 92 North Second Rd, Shenyang, 110001 Liaoning Province China; 2 The First People’s Hospital of Shenyang, Shenyang City, 110041 Liaoning Province China; 3 Department of Neurology, The Fourth Affiliated Hospital of China Medical University, Shenyang City, 110032 Liaoning Province China

**Keywords:** Endothelial dysfunction, Mild stroke, Cystatin C, Glomerular filtration rate, Post stroke cognitive impairment, Medical research, Neurology

## Abstract

The incidence of post stroke cognitive impairment (PSCI) is high in patients with mild stroke (MIS), and the risk factors and mechanism are uncertain. Increased cystatin C (CysC) levels after stroke may reflect lower glomerular filtration rate (GFR) and renal impairment. Previous studies have suggested endothelial dysfunction (ED) is closely related to renal impairment and cognitive impairment, respectively. We aimed to observe whether lower GFR estimated by CysC after MIS leaded to a high incidence of PSCI, and the role of ED in this process. 256 patients were enrolled in this prospective observational study. Renal function was assessed using GFR estimated by serum CysC. Endothelial function was evaluated by reactive hyperemia index (RHI) which calculated automatically by peripheral arterial tonometry (PAT). The cognitive function at baseline and 3 months was evaluated by MoCA score, and MoCA score ≤ 26 indicates the presence of PSCI. Spearman correlation analysis and linear regression were conducted to explore the factors affecting ED. Univariate and multivariate analysis was used to identify the independent risk factors of PSCI. The receiver operating characteristic (ROC) curve was applied to explore the optimal cutoff value of the independent risk factors levels for predicting PSCI. A total of 141 patients (55.1%) suffered from ED. Multiple linear regression analysis showed that there was a strong linear correlation between eGFRcys and RHI (p < 0.001). At the three-month follow-up, a total of 150 (58.6%) patients had been diagnosed with PSCI. Multivariate logistic regression analysis showed that RHI was an independent factor affecting the occurrence of PSCI (p < 0.05). ROC curve showed that the area under the curve was 0.724, and the optimal cut-off value of RHI was 1.655, with the sensitivity and specificity for PSCI were 72.7% and 73.6%, respectively. The lower eGFRcys level after MIS was significantly associated with ED, and ED may mediate the higher incidence of PSCI at 3 months after MIS.

## Introduction

More than half of patients with acute ischemic stroke (AIS) have mild symptoms at the initial presentation, among which, patients with NIHSS < 5 were defined as MIS^1^. And these patients are often ignored because of less limb paralysis and fast recovery^2^. Some studies show that the incidence rate of post-stroke cognitive impairment (PSCI) is high in patients with acute mild stroke (MIS), and long-term studies of MIS showed that impaired concentration, memory, and overall reduced quality of life were quite common despite lack of motor impairments or global cognitive screening abnormalities^3^. Similarly, more than half of patients of MIS with good functional recovery still have cognitive impairment^4^. Therefore, it is urgent to prevent PSCI in patients with MIS. However, studies on the risk factors of cognitive dysfunction in MIS are rare, and further researches are needed.

Glomerular filtration rate (GFR) is considered as the best marker to determine renal function^5^. In recent years, because creatinine (Cr) is affected by muscle quality, gender, race and diet factors, and particularly, mild and moderate declines in renal function are inaccurately displayed by GFR estimated by creatinine, cystatin C (CysC) has been considered as an alternative biomarker for GFR evaluation^6^. CysC is expressed in all nucleated cells of the human body, and can be freely filtered in the glomeruli. The secretion of CysC in serum was constant, and as a new biomarker of glomerular function, it has attracted more and more attention^7^.Previous studies have reported that even mild renal function damage may have a poor prognosis for patients with AIS^8^. Meanwhile, lots of studies have reported that the level of serum CysC increased significantly after stroke^9,10^. However, few scholars associated the elevated level of serum CysC with early renal dysfunction. And early detection of renal dysfunction and intervention may play a positive role in the prognosis of stroke.

Endothelial dysfunction (ED), one of the first steps in the development of atherosclerosis, is also an early manifestation of renal function damage^11^. And ED is also closely related to microcirculation disorders^12^. Meanwhile, ED can induce destruction of blood brain barrier and the damage of neurons^13^. Furthermore, it has been reported that ED is also closely related to cognitive impairment^14^. Therefore, ED may be the common mechanism of renal function damage and cognitive impairment.

We designed this prospective study to investigate whether lower levels of GFR estimated by cystatin C after MIS are associated with ED and whether they can lead to a higher incidence rate of PSCI.

## Methods

### Study Population

This study was approved by the Ethics Committee of the First Affiliated Hospital of China Medical University (Ethics No.: 2021-457). All research processes followed the declaration of Helsinki. We prospectively collected the acute MIS patients hospitalized in the First Affiliated Hospital of China Medical University from January 2021 to December 2021. We used the National Institutes of Health Stroke Scale (NIHSS) to distinguish the severity of stroke, and defined patients with NIHSS score less than 5 as MIS^15^.

The admission criteria were: (1) age ≥ 18 years; (2) the onset time ≤ 3 days; (3) patients with AIS diagnosed by diffusion weighted imaging (DWI) for the first time; (4) NIHSS score < 5; (5) agree to participate in the study and sign the written informed consent. Participants were excluded based on: (1) patients with cognitive impairment at admission identified by a MOCA score < 27; (2) patients with previous diagnosis of central nervous system diseases: (3) patients with tumors; (4) patients unable to complete the scale or related examinations due to hearing, vision or language disorders; (5) complicated course with serious heart disease, such as acute myocardial infarction, acute left heart failure, etc. (6) patients receiving intravenous thrombolysis (IVT).

Demographics and basic clinical information were registered at admission. The severity of neurological function after stroke was assessed by NIHSS score, the etiology classification of stroke was assessed by Trial of Org 10172 in Acute Stroke Treatment(TOAST classification), and the baseline cognitive function was assessed by Montreal Cognitive Assessment (MoCA). Fasting after 22:00 on the day of admission, blood samples were collected in the morning of the next day. And the blood samples were sent to the Laboratory Department of the First Affiliated Hospital of China Medical University for measurement by an automatic blood analyzer. The reference value of serum CysC ranged from 0.53 to 0.95 mg/l. Intra- and inter-assay coefficients of variation were less than 3.9% and 4.8%, respectively. Blood pressure of all the patients needed to be well controlled, and the blood pressure should be controlled at about 140/90 mmHg, with a range of less than 20 mmHg.

### Kidney function

Renal function was assessed by GFR estimated by serum CysC and creatinine levels. We used the Chronic Kidney Disease Epidemiology Collaboration Equation (CKD-EPI) to calculate GFR levels, expressed as eGFRcr (GFR calculated by serum Cr, 2009 equation for Cr) and eGFRcys (GFR calculated by serum CysC, 2012 equation for CysC), respectively^7,16^. GFR ranging from 61 to 90 mL/min/1.73 m^2^ was considered as mild renal function damage, which was defined as CKD Stages 1–2, and GFR < 60 mL/min/1.73 m^2^ was renal function damage, which was defined as CKD Stages 3–5^8^.

### Endothelial function

Endothelial function was evaluated by reactive hyperemia index (RHI) which calculated automatically by peripheral arterial tonometry (PAT). And we completed the examination within one week after the onset of stroke. The examination should be conducted in a quiet and appropriate temperature environment. The endothelial mediated changes in vascular tension were quantified by occluding the brachial artery for 5 min with standard cuff inflation. When the cuff deflates, the blood flow shocks cause reactive congestion, which can be captured by RHI-PAT in time, and the signal amplitude ratio before and after blocking can be calculated by software to obtain the evaluation index of endothelial function RHI. RHI < 1.67 was considered to indicate ED^17^.

### Assessment of cognitive function

The cognitive function of the patients at baseline and 3 months was evaluated by using the MoCA scale. The evaluation process was conducted by trained neurologists. The total score of MoCA is 30 points, and the lower the score, the more serious of the PSCI in patients with MIS. Cognitive function is classified as follows: 27–30 points is defined as no cognitive impairment, and 0–22 points is defined as post-stroke dementia (PSD). In this study, MoCA score ≤ 26 indicates the presence of PSCI.

### Statistical analysis

The continuous variables were expressed as mean ± standard deviation (SD), and t-test was performed for independent group comparison. The categorical variables were expressed as count (%), and were analyzed by χ^2^ test. Correlation analysis between the two variables was conducted using Spearman correlation analysis, and the influencing factors were analyzed by Logistic regression analysis and linear regression. The levels of each index between PSCI and without PSCI groups were compared to find out the influencing factors of PSCI in univariate analysis. Logistic regression analysis was used to identify the independent risk factors for the presence of PSCI within variables with p < 0.05 in the univariate analysis. The receiver operating characteristic (ROC) curve was applied to explore the optimal cutoff value of the independent risk factors levels for predicting PSCI. For all the analyses, P < 0.05 was considered statistically significant. Statistical analyses had been carried out by the use of the SPSS program (Version 21.0, IBM Statistics). Results are reported according to the STROBE reporting guidelines (Supplemental Material)^18^.

### Ethical approval and consent to participate

This study involving human participants were reviewed and approved by the Regional Medical Scientific Research Ethics Committee of the First Affiliated Hospital of China Medical University (IRB no. 2021457).

## Results

**Figure 1:**
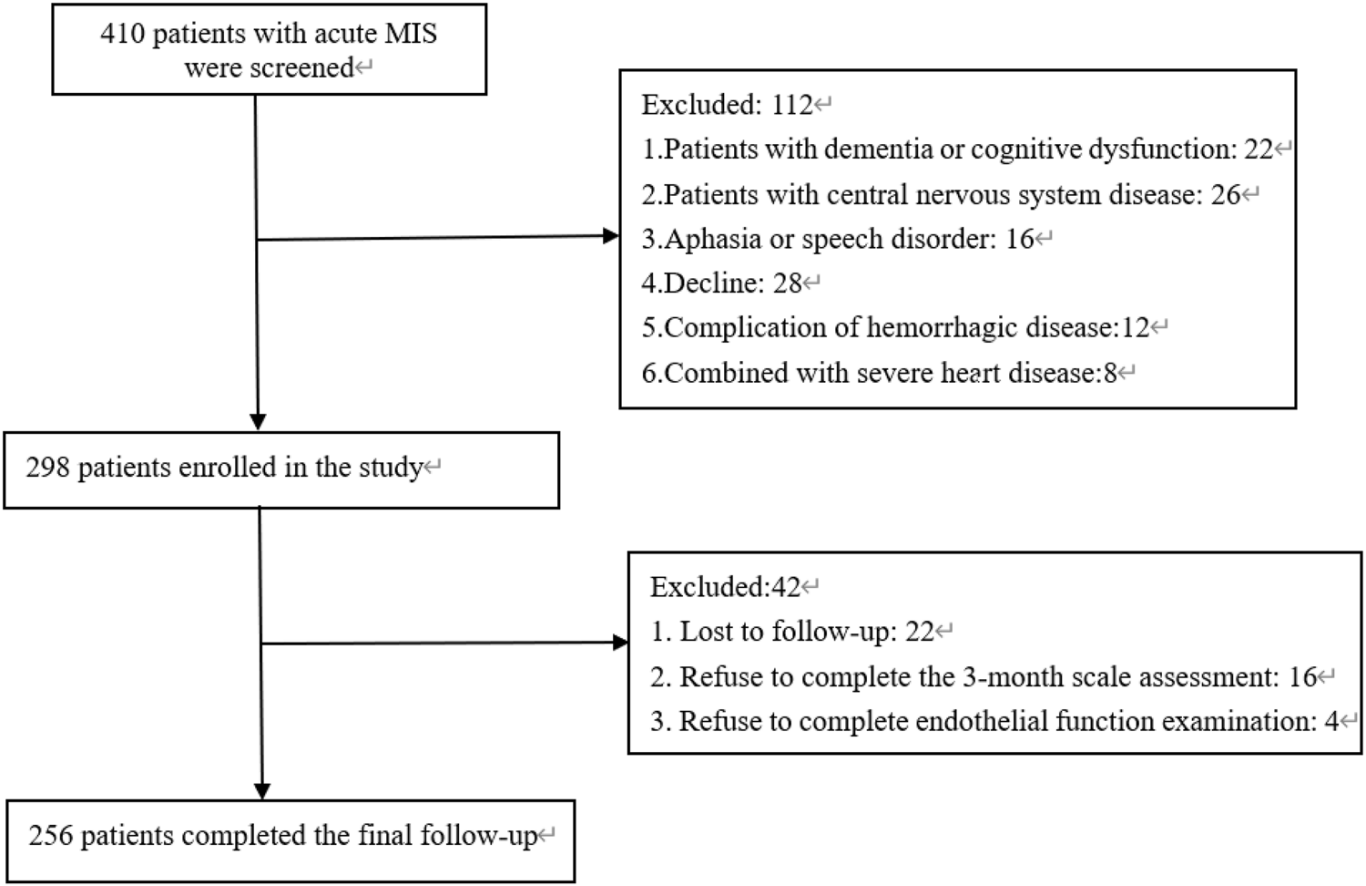
Study recruitment process.

From January 2021 to December 2021, we prospectively sequentially screened 410 patients with acute MIS. A total of 298 patients met the inclusion and exclusion criteria and were included in this study. Among the 298 patients, a total of 42 patients did not complete the final follow-up, including 22 patients who were lost to follow-up, 16 patients who refused to complete the relevant follow-up scale, and 4 patients who refused to complete the endothelial function test. Therefore, 256 patients were eventually enrolled in the study. Figure 1 shows the detailed process. The demographic and clinical characteristics of the 256 patients are shown in Table 1. The mean CysC levels were 1.06 mg/l, significantly higher than the reference range (0.53 to 0.95 mg/l). The mean eGFRcr levels were 95.50 mL/min per 1.73 m^2^, significantly higher than eGFRcys, which was just 74.54 mL/min per 1.73 m^2^. The MoCA score at baseline was 27.69 ± 0.75. Additionally, the mean RHI was 1.76 ± 0.44.

**Table 1:** Population characteristics.

Variables	Overall (n = 256)
Sex (male)	176 (68.8)
Age (year)	60.73 ± 12.46
Years of education (year)	8.18 ± 4.72
Current smoking (n, %)	94 (36.7)
Current drinking (n, %)	48 (18.8)
Hypertension (n, %)	160 (62.5)
Systolic blood pressure(mmHg)	153.21 ± 21.49
Diastolic blood pressure(mmHg)	87.98 ± 14.78
Cardiovascular disease (n, %)	34 (13.3)
Atrial fibrillation(n, %)	10 (3.9)
Diabetes (n, %)	79 (30.9)
Time from onset to randomization(hour)	31.07 ± 19.08
NIHSS at admission	1.93 ± 1.20
Stroke etiology, n (%)	
Atherosclerosis	95 (37.1)
Cardio embolism	7 (2.7)
Small vessel occlusion	134 (52.3)
Other undetermined etiology	3 (1.2)
Undefined	17 (6.6)
MoCA score	27.69 ± 0.75
Laboratory variables	
Urea nitrogen, mmol/L	5.77 ± 5.47
eGFRcr, mL/min per 1.73 m^2^	95.50 ± 15.84
eGFRcr ≥ 90	189 (73.8)
eGFRcr < 90	67 (26.2)
eGFRcys, mL/min per 1.73 m^2^	74.54 ± 18.68
eGFRcys ≥ 90	54 (21.1)
eGFRcys < 90	202 (78.9)
Creatinine, umol/L	67.17 ± 19.85
HDL-cholesterol, mmol/L	1.09 ± 0.31
Triglyceride, mmol/L	1.63 ± 0.91
Total cholesterol, mmol/L	4.49 ± 1.09
LDL-cholesterol, mmol/L	2.86 ± 0.90
Glycated hemoglobin, %	6.59 ± 1.70
Folic acid, nmol/L	17.30 ± 9.48
HCY, umol/L	14.71 ± 9.10
CysC, mg/L	1.06 ± 0.24
RHI	1.76 ± 0.44

NIHSS, National Institute of Health Stroke Scale; GFR, glomerular filtration rate; HDL, high-density lipoprotein; LDL, low-density lipoprotein; HCY,homocysteine; MoCA, Montreal Cognitive Assessment; CysC, Cystatin C; RHI, reactive hyperemia-peripheral arterial tonometry.

**Figure 2:**
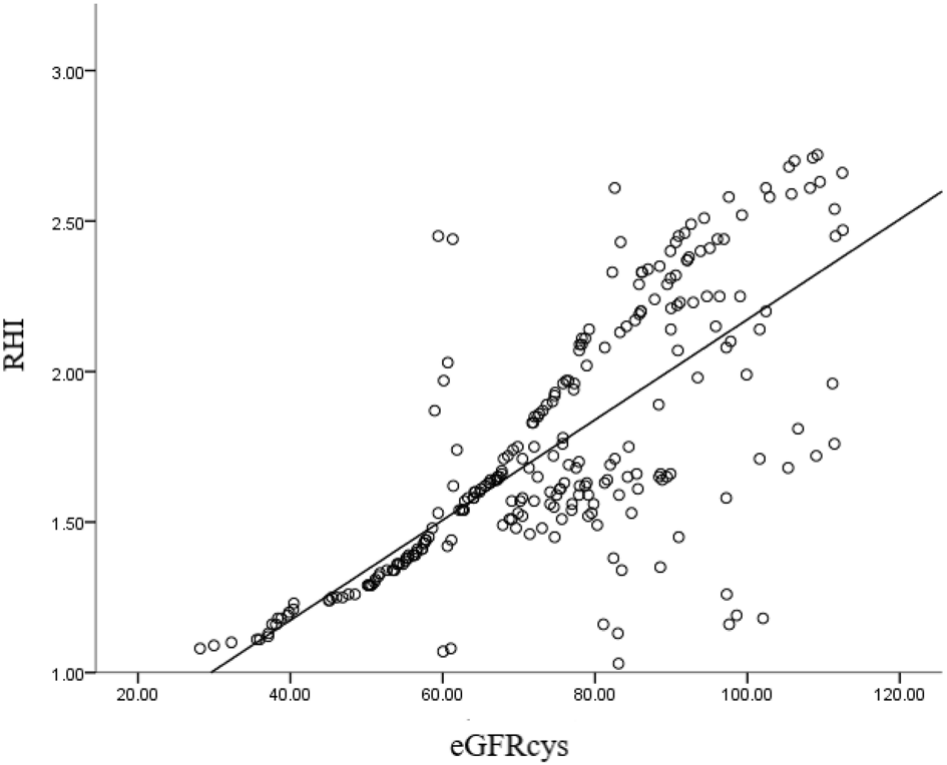
The correlation between eGFRcys and RHI.

Table 2 shows the correlation between RHI and various influencing factors, and found that higher age, years of education, combined with diabetes, increased uric acid, increased urea nitrogen, increased creatinine, increased homocysteine(HCY), increased CysC, decreased eGFRcys and decreased eGFRcr significantly correlated with RHI(p < 0.05). As shown in Table3, a total of 141 patients (55.1%) suffered from ED. eGFRcr > 90 mL/min per 1.73 m^2^ was found in 189patients, and a total of 83 (43.9%) patients had ED, while eGFRcys > 90 mL/min per 1.73 m^2^ was found in 54 patients, and only 6 (11.1%) patients had ED. Multiple linear regression analysis showed that there was a strong linear correlation between eGFRcys and RHI, that is, the lower the eGFRcys levels, the lower RHI index (p < 0.001, Table 4, Fig. 2).

**Table 2:** Correlation analysis between RHI and various influencing factors.

Spearman	N	R	P
Sex (male)	256	0.122	0.052
Age (year)	256	−0.267	< 0.001
Years of education (year)	256	0.198	0.001
Current smoking (n, %)	256	−0.059	0.345
Current drinking (n, %)	256	−0.024	0.706
Hypertension (n, %)	256	−0.070	0.261
Cardiovascular disease (n, %)	256	0.062	0.321
Diabetes (n, %)	256	0.145	0.020
Atrial fibrillation (n, %)	256	−0.032	0.613
Time from onset to randomization (hour)	256	0.045	0.474
NIHSS at admission	256	−0.048	0.447
MoCA scoreat admission	256	−0.019	0.760
MRSat admission	256	−0.014	0.827
Uric Acid,umol/L	256	−0.268	< 0.001
Urea nitrogen, mmol/L	256	−0.410	< 0.001
Creatinine, umol/L	256	−0.443	< 0.001
HDL-cholesterol, mmol/L	256	0.110	0.080
Triglyceride, mmol/L	256	0.020	0.746
Total cholesterol, mmol/L	256	0.006	0.928
LDL-cholesterol, mmol/L	256	−0.025	0.691
Glycated hemoglobin, %	256	0.075	0.231
Folic acid, nmol/L	256	0.111	0.076
HCY, umol/L	256	−0.266	< 0.001
Hemoglobin,g/L	256	0.055	0.378
CysC, mg/L	256	−0.722	< 0.001
eGFRcys, mL/min per 1.73 m^2^	256	0.733	< 0.001
eGFRcr, mL/min per 1.73 m^2^	256	0.539	< 0.001

NIHSS, National Institute of Health Stroke Scale; GFR, glomerular filtration rate; HDL, high-density lipoprotein; LDL, low-density lipoprotein; HCY,homocysteine; MoCA, Montreal Cognitive Assessment; CysC, Cystatin C; RHI, reactive hyperemia-peripheral arterial tonometry.

**Table 3:** Comparison of different glomerular filtration rate levels with RHI.

		RHI		X^2^	P
		≥ 1.67 (N = 115)	< 1.67 (N = 141)		
eGFRcys	< 60	2 (3.4)	57 (96.6)	83.339	< 0.001
	60 ≤ eGFRcys < 90	65 (45.5)	78 (54.5)		
	≥ 90	48 (88.9)	6 (11.1)		
eGFRcr	< 60	0 (0.0)	7 (100.0)	42.317	< 0.001
	60 ≤ eGFRcr < 90	9 (15.0)	51 (85.0)		
	≥ 90	106 (56.1)	83 (43.9)		

GFR, glomerular filtration rate; RHI, reactive hyperemia-peripheral arterial tonometry.

**Table 4:** Multiple linear regression analysis affecting RHI level.

	B	S.E	β	t	*P*	95.0% CI	
Normal	−0.001	0.726		−0.002	0.998	−1.432	1.429
Age	0.002	0.002	0.067	0.923	0.357	−0.003	0.007
Years of education	−0.002	0.004	−0.027	−0.594	0.553	−0.01	0.006
Diabetes	0.049	0.041	0.053	1.182	0.238	−0.032	0.13
Uric Acid	−0.0004	0.0003	−0.078	−1.56	0.120	−0.001	0.0001
Urea nitrogen	−0.003	0.003	−0.036	−0.825	0.410	−0.01	0.004
Creatinine	0.002	0.002	0.091	0.899	0.370	−0.002	0.006
HCY	−0.002	0.002	−0.049	−1.02	0.309	−0.007	0.002
CysC	0.131	0.293	0.074	0.448	0.655	−0.446	0.709
eGFRcys	0.017	0.004	0.761	4.884	< 0.001	0.01	0.024
eGFRcr	0.002	0.003	0.092	0.904	0.367	−0.003	0.008

GFR, glomerular filtration rate; HCY, homocysteine; CysC, Cystatin C; RHI, reactive hyperemia-peripheral arterial tonometry.

At the three-month follow-up, a total of 150 (58.6%) patients had been diagnosed with PSCI. Table 5 describes the univariate analysis of variables between the PSCI and without PSCI group. Age, diabetes, uric acid, creatinine, CysC, RHI, HCY, eGFRcys and eGFRcr were all risk factors for the occurrence of PSCI (p < 0.05). Multivariate logistic regression analysis showed that RHI was an independent factor affecting the occurrence of PSCI (p < 0.05). And the other factors were not independent risk factors of PSCI (p > 0.05, Table 6).

**Table 5:** Univariate analysis between PSCI and without PSCI group.

	Without PSCI N = 106	PSCI N = 150	t /X^2^	P
Sex (male)	68(64.2)	108(72.0)	1.781	0.182
Age (year)	58.75 ± 10.82	62.13 ± 13.35	−2.148	0.033
Years of education (year)	8.18 ± 1.41	8.18 ± 6.07	−0.001	0.999
Current smoking (n, %)	37(34.9)	57(38.0)	0.256	0.613
Current drinking (n, %)	17(16.0)	31(20.7)	0.874	0.350
Hypertension (n, %)	68(64.2)	92(61.3)	0.210	0.646
Cardiovascular disease (n, %)	19(17.9)	15(10)	5.185	0.023
Diabetes (n, %)	41(38.7)	38(25.3)	0.521	0.471
Atrial fibrillation (n, %)	4(3.8)	6(4.0)	0.008	0.927
Time from onset to randomization (hour)	32.25 ± 19.76	30.23 ± 18.61	0.837	0.403
NIHSS at admission	1.80 ± 1.23	2.01 ± 1.17	−1.394	0.164
MoCA scoreat admission	27.66 ± 0.74	27.71 ± 0.76	−0.483	0.629
Stroke etiology, n (%)			4.321	0.364
Atherosclerosis	32(30.2)	63(42)		
Cardio embolism	4(3.8)	3(2)		
Small vessel occlusion	61(57.5)	73(48.7)		
Other undetermined etiology	1(0.9)	2(1.3)		
Undefined	8(7.5)	9(6)		
Uric Acid,umol/L	303.94 ± 80.21	325.66 ± 73.38	−2.244	0.026
Urea nitrogen, mmol/L	6.20 ± 8.27	5.46 ± 1.66	1.076	0.283
Creatinine, umol/L	61.77 ± 15.23	70.99 ± 21.80	−3.751	< 0.001
HDL-cholesterol, mmol/L	1.11 ± 0.32	1.08 ± 0.30	0.589	0.556
Triglyceride, mmol/L	1.70 ± 0.99	1.59 ± 0.86	0.927	0.355
Total cholesterol, mmol/L	4.53 ± 1.16	4.46 ± 1.05	0.516	0.606
LDL-cholesterol, mmol/L	2.88 ± 0.91	2.85 ± 0.90	0.296	0.767
Glycated hemoglobin, %	6.78 ± 1.76	6.47 ± 1.66	1.432	0.153
Folic acid, nmol/L	18.17 ± 9.91	16.69 ± 9.14	1.239	0.217
HCY, umol/L	13.22 ± 8.33	15.76 ± 9.50	−2.217	0.028
Hemoglobin,g/L	143.42 ± 18.87	143.85 ± 15.19	−0.202	0.840
CysC, mg/L	0.96 ± 0.17	1.13 ± 0.26	−5.827	< 0.001
eGFRcys, mL/min per 1.73 m^2^	82.8 ± 16.33	68.71 ± 18.07	6.394	< 0.001
eGFRcr, mL/min per 1.73 m^2^	100.24 ± 12.11	92.15 ± 17.28	4.156	< 0.001
RHI	1.93 ± 0.41	1.62 ± 0.39	6.234	< 0.001

NIHSS, National Institute of Health Stroke Scale; GFR, glomerular filtration rate; HDL, high-density lipoprotein; LDL, low-density lipoprotein; HCY,homocysteine; MoCA, Montreal Cognitive Assessment; CysC, Cystatin C; PSCI, post stroke cognitive impairment;RHI, reactive hyperemia-peripheral arterial tonometry.

**Table 6:** Logistic regression analysis for the occurrence of PSCI.

	B	S.E	Wald	P	OR	OR 95% C.I	
Age	0.016	0.019	0.692	0.406	1.016	0.978	1.056
Diabetes	−0.435	0.31	1.977	0.160	0.647	0.353	1.187
Uric Acid	0.0004	0.002	0.046	0.830	1.0004	0.996	1.005
Creatinine	0.006	0.018	0.126	0.723	1.006	0.972	1.042
HCY	0.005	0.018	0.069	0.793	1.005	0.969	1.041
CysC	1.648	3.005	0.301	0.583	5.196	0.014	1.876
eGFRcys	−0.012	0.034	0.131	0.718	0.988	0.925	1.055
GFRcr	0.014	0.021	0.45	0.502	1.014	0.973	1.058
RHI	−0.944	0.46	4.21	0.040	0.389	0.158	0.959

GFR, glomerular filtration rate; HCY, homocysteine; CysC, Cystatin C; RHI, reactive hyperemia-peripheral arterial tonometry.

**Figure 3:**
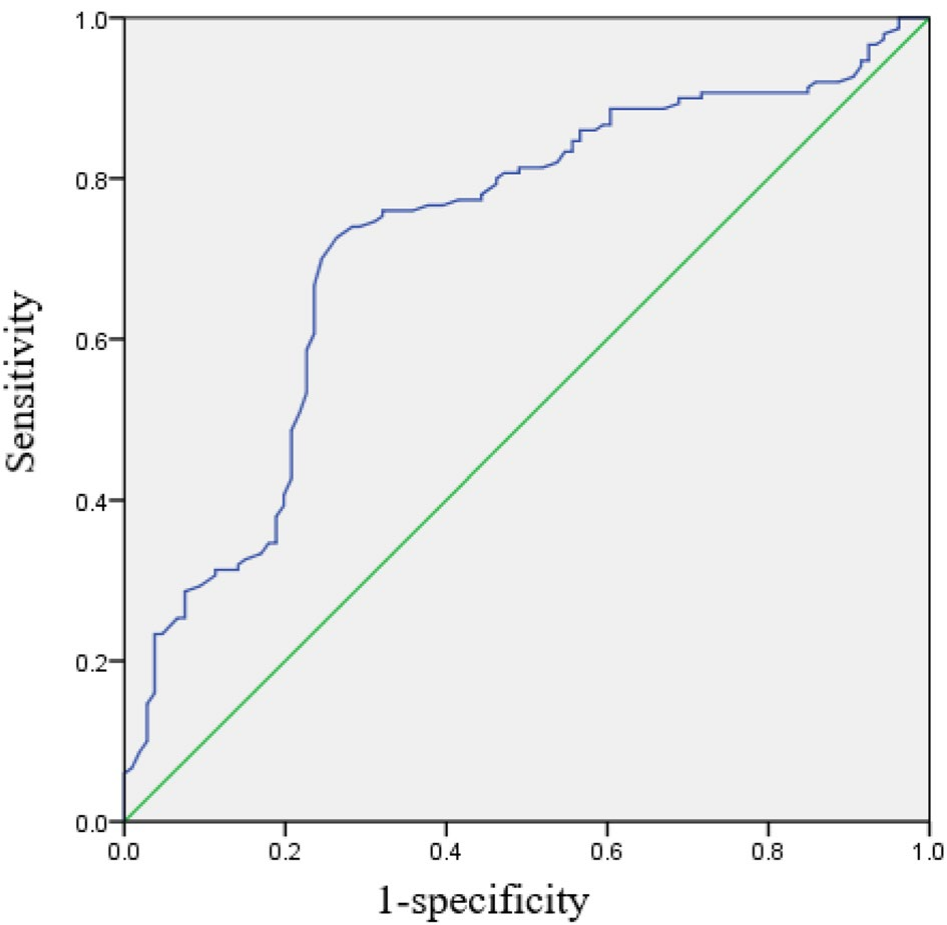
The ROC curve to detect PSCI.The best cut-off of RHI to detect PSCI was 1.655. The AUC was 0.724 (p < 0.001).

The accuracy of RHI in predicting PSCI was evaluated by ROC curve. The area under the curve (AUC) was 0.724. The optimal cut-off value of RHI was 1.655, and the sensitivity and specificity for PSCI were 72.7% and 73.6%, respectively (95% CI 0.66–0.789, p < 0.001, Fig. 3).

## Discussion

Our study assessed endothelial function by RHI-PAT, and found that more than half of patients with MIS had ED after stroke. We also found that there was a strong linear correlation between eGFRcys and RHI, and the lower the eGFRcys levels, the lower RHI index, which can better predict the occurrence of ED. At the same time, RHI was an independent factor affecting the occurrence of PSCI. Therefore, ED may mediate the higher incidence rate of PSCI in patients with lower GFR after MIS.

MIS is a kind of stroke with mild symptoms and less paralysis. At present, about two-thirds of patients with AIS only experience less neurological dysfunction during the acute phase^19^. Previous studies have shown that patients with MIS have a high incidence rate of PSCI^20^. Similarly, our study excluded patients with cognitive impairment prior to enrollment and found that the incidence of PSCI was also more than 50%. The patients with MIS often do not pay attention to the prevention and treatment after stroke, and seldom cooperate with follow-up after discharge, which makes it difficult to identify and prevent PSCI. Therefore, early detection of the risk factors affecting the occurrence of PSCI, and early intervention for these patients, may receive good results.

CysC is a low molecular weight non-glycosylated basic protein^21^.Previous studies have shown that serum CysC is independent of muscle mass and is more strongly associated with kidney function than creatinine^22^. And CysC may be superior to creatinine in early detection of kidney injury^23^. Likewise, GFR estimated by creatinine less than 90 mL/min per 1.73 m^2^ was just found in only 67(26.2%) of patients in our study, which was significantly lower than that of CysC, and the results were similar to those of other studies^8^. In recent years, GFR and renal injury assessed by CysC have received increasing attention^24,25^. Studies have reported that eGFRcys is associated with tumors, subclinical atherosclerosis and coronary artery calcification^26,27^. However, few studies have focused on changes in eGFRcys after stroke and its association with stroke prognosis. In our study, we found that the mean level of CysC after MIS was significantly higher than the reference range of normal values, and so the GFR level assessed by CysC decreased significantly. Does this change result in a poor prognosis for MIS patients? In our study, we found that there was a strong linear correlation between eGFRcys and RHI, and therefore, lower eGFRcys levels after MIS can reflect impairment of endothelial function.

Endothelium is a crucial organ that regulates the vascular system to meet physiological needs, and ED is a pathological condition of cardiovascular and cerebrovascular diseases^28,29^. Especially when the renal function decreases, it may be accompanied with ED and microvascular dysfunction^30^. Moreover, ED is an early feature of cerebral small vessel disease, which has prognostic significance for cerebrovascular disease in vascular event recurrence and vascular cognitive impairment^31^. Therefore, the occurrence of PSCI may be closely related to ED^32^.In our study, we explored the correlation between ED after MIS and cognitive impairment at 3 months, and found RHI was an independent factor affecting the occurrence of PSCI. At the same time, the area under the curve for the diagnosis of PSCI is more than 0.7, and it also has high sensitivity and specificity. This means that early detection and intervention of ED may reduce the incidence of PSCI. However, the examination of endothelial function is not popular now, and many hospitals cannot complete the relevant examination in time. Our study found that patients with low GFR are closely related to the occurrence of ED. As a result, early intervention and improved examination of endothelial function in these patients may be more effective.

There were some limitations of the present study. First, this study was a single-center observational study, and although we collected all patients with MIS who met the inclusion criteria within a whole year, further multicenter studies are needed to confirm our conclusions. Second, eGFRcys and RHI were recorded only once and we cannot capture changes in RHI with the decline of renal function. Finally, the follow-up period was relatively short.

## Conclusion

Our study explored the risk factors of PSCI and its internal mechanism in patients with MIS. The current research results showed that lower eGFRcys level after MIS was significantly associated with poor endothelial function, and ED may mediate the occurrence of PSCI.

## Data Availability

Data are available from the corresponding author on reasonable request.
