# Author Correction: Lower glomerular filtration rate after mild stroke induces cognitive impairment by causing endothelial dysfunction

**DOI:** 10.1038/s41598-024-70730-x

**Published:** 2024-08-26

**Authors:** Xu Yan, Huan Chen, Xiuli Shang

**Affiliations:** 1 Department of Neurology, The First Affiliated Hospital of China Medical University, 92 North Second Rd, Shenyang, 110001 Liaoning Province China; 2 The First People’s Hospital of Shenyang, Shenyang City, 110041 Liaoning Province China; 3 Department of Neurology, The Fourth Affiliated Hospital of China Medical University, Shenyang City, 110032 Liaoning Province China

Correction to: *Scientific Reports* 10.1038/s41598-024-57444-w, published online 23 March 2024

The original version of this Article contained an error in the order of the author names, which was incorrectly given as Xiuli Shang, Xu Yan, and Huan Chen.

The Author contributions section remains the same.

The original Article has been corrected.

